# Liver metastasis composed of pure squamous cell carcinoma component from pancreatic pure ductal adenocarcinoma: a case report

**DOI:** 10.1186/s40792-023-01755-z

**Published:** 2023-09-29

**Authors:** Yohei Mano, Keishi Sugimachi, Tomonari Shimagaki, Takahiro Tomino, Emi Onishi, Lingaku Lee, Terumasa Hisano, Yutaka Koga, Kenichi Taguchi, Masaru Morita, Yasushi Toh

**Affiliations:** 1 Department of Hepato-Biliary and Pancreatic Surgery, National Hospital Organization Kyushu Cancer Center, Notame 3-1-1, Minami-ku, Fukuoka, 811-1395 Japan; 2 Department of Surgery, Oita Red Cross Hospital, 3-2-37 Chiyomachi, Oita, 870-0033 Japan; 3 Department of Hepato-Biliary-Pancreatology, National Hospital Organization Kyushu Cancer Center, Notame 3-1-1, Minami-ku, Fukuoka, 811-1395 Japan; 4 Department of Pathology, National Hospital Organization Kyushu Cancer Center, Notame 3-1-1, Minami-ku, Fukuoka, 811-1395 Japan; 5 Department of Gastroenterological Surgery, National Hospital Organization Kyushu Cancer Center, Notame 3-1-1, Minami-ku, Fukuoka, 811-1395 Japan

**Keywords:** Pancreatic cancer, Squamous cell carcinoma, Liver metastasis, *KRAS* mutation

## Abstract

**Background:**

Liver metastasis of pure squamous cell carcinoma (SCC) from pancreatic ductal adenocarcinoma has not been previously reported.

**Case presentation:**

A 66-year-old man underwent a computed tomography scan 3 years after surgery for pancreatic head cancer, and the scan revealed a mass lesion in the right lobe of the liver. A liver tumor biopsy was performed, and SCC was diagnosed. Whole sections of the pancreatic head cancer were re-evaluated, but no areas of SCC-like differentiation were identified. Although the pathology differed between the pancreas and liver, metastasis of adenosquamous carcinoma was considered. Three courses of gemcitabine plus nab-paclitaxel were administered to treat the liver metastasis of pancreatic cancer, but no response was attained. Therefore, primary SCC of the liver was considered and hepatic resection was performed. The tumor had invaded the diaphragm, and S5/6 partial hepatic resection with right diaphragm resection was performed. Pathological examination showed pure SCC of the liver, which differed from the pancreatic cancer. *KRAS* mutations were evaluated in the pancreatic and liver tumor specimens, and *Q61R* mutation was identified in both specimens. This pure SCC of the liver was diagnosed as metastasis from pancreatic cancer not by histology but by genetic analysis.

**Conclusions:**

This is the first reported case of pure SCC liver metastasis from pancreatic cancer without a squamous cell component in the primary tumor. Evaluation of *KRAS* mutations in both specimens was useful for diagnosis.

## Background

Pancreatic cancer is a malignancy with a poor prognosis. Pathological phenotypes of pancreatic cancer include adenocarcinoma, adenosquamous carcinoma (ASC), and squamous cell carcinoma (SCC). Primary pancreatic SCC is extremely rare and has a poor prognosis [1–3]. We herein report a case in which pure SCC of the liver appeared after surgery for adenocarcinoma of the pancreatic head, and the homology of *KRAS* mutation revealed that the liver tumor was a liver metastasis of the pancreatic cancer.

## Case presentation

**Fig. 1:**
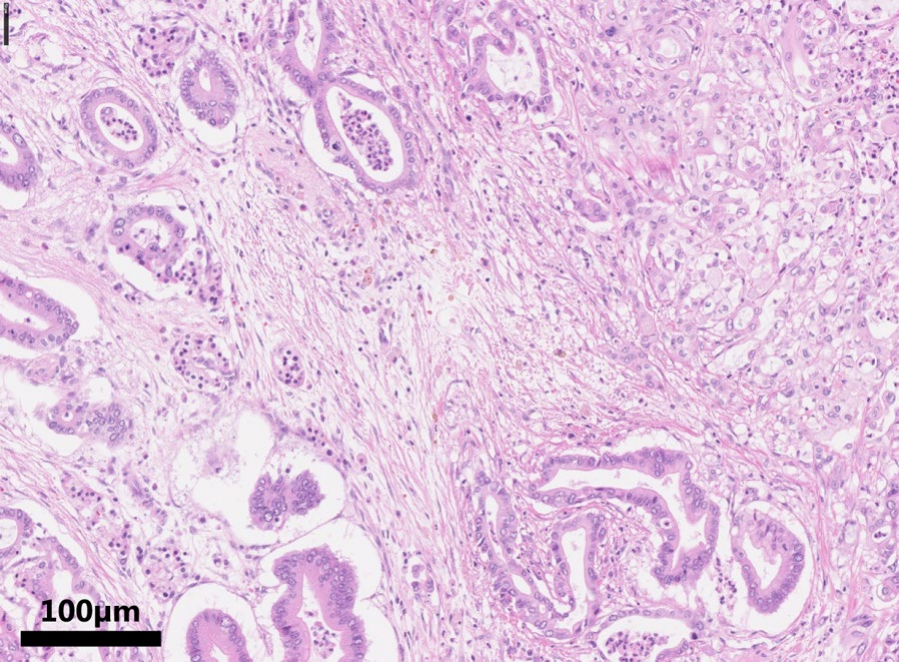
Pathological findings of the previous pancreatic cancer. Heteromorphic cells formed glandular tubular structures with a fibrous stroma. No squamous cell carcinoma component was observed

**Fig. 2:**
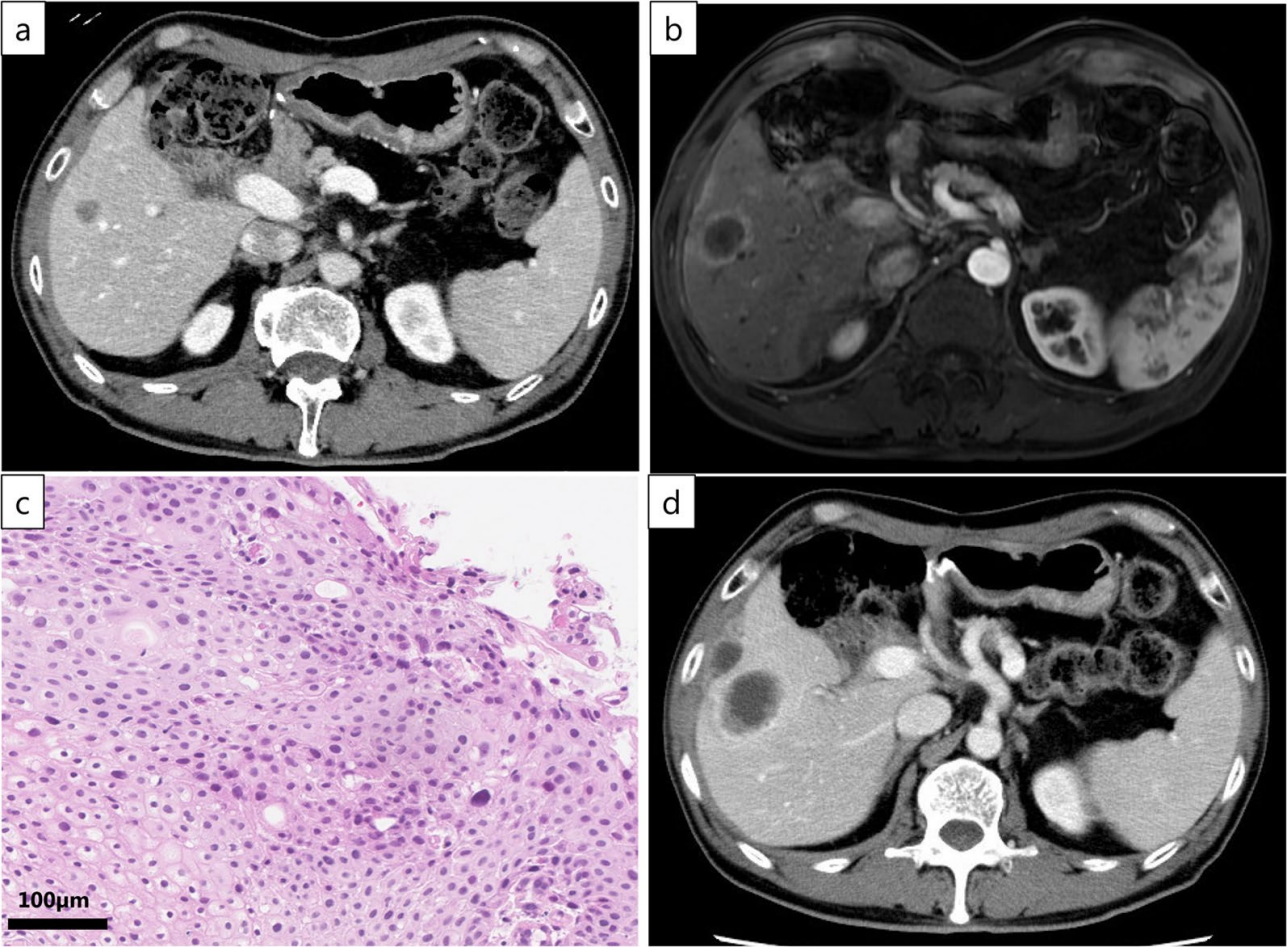
Computed tomography (CT) scan, magnetic resonance imaging (MRI) and pathological findings of the liver tumor. **a** Three years after pancreatectomy, CT revealed a 3-cm tumor lesion in the right lobe of the liver. **b** The tumor was depicted on gadoliniumethoxybenzyl diethylene triaminepentaacetic acid-MRI as enhanced peritumor in the early phase. **c** Atypical cells with large nuclei were growing in sheets, suggesting squamous cell carcinoma. **d** Three months later, CT showed that the liver tumor had grown to 6 cm in diameter

A 66-year-old man underwent pancreatoduodenectomy 3 years previously for treatment of pancreatic head cancer. Pathological examination of the excised specimen revealed pure pancreatic adenocarcinoma with no lymph node metastasis (Fig. 1). The pathological diagnosis at that time was T2N0M0 Stage IB according to the UICC TNM classification. The tumor was 1.4 × 1.0 cm in diameter, with invasion into the duodenum. Lymph node metastasis and invasion into the perineurium, plexus, portal vein, arteries, or other surrounding organs were not observed, but lymphatic and venous invasion around the tumor was observed. Postoperatively, the patient underwent periodic imaging and blood tests to search for recurrence. Three years after pancreatoduodenectomy, computed tomography (CT) revealed a 3-cm tumor lesion in the right lobe of the liver (Fig. 2a). The tumor showed low signal on T1-weighted magnetic resonance imaging (MRI) and high signal on T2-weighted MRI. The tumor was depicted on gadoliniumethoxybenzyl diethylene triaminepentaacetic acid-MRI as enhanced peritumor in the early phase and as deficient in the hepatobiliary phase (Fig. 2b). The enhancement dynamics were similar to those of metastatic liver tumors such as adenocarcinoma. Liver tumor biopsy showed atypical cells with large nuclei growing in sheets, and the diagnosis was SCC (Fig. 2c). Immunohistochemical staining showed that the atypical cells were positive for p40 and CK19, but negative for CK7. A search of organs in which SCC might occur (head and neck, esophagus, skin, and anus) revealed no obvious primary lesion. Because primary SCC of the liver is extremely rare, the liver tumor was considered a metastasis of the pancreatic cancer. The patient underwent three courses of combination therapy of gemcitabine plus nab-paclitaxel. The disease was evaluated by CT, which showed that the tumor had increased in size and that the disease was progressive (Fig. 2d). Therefore, the patient was treated with a combination of carboplatin and paclitaxel, which is expected to be effective for SCC. Before the efficacy could be confirmed, the patient was referred to our hospital at his request.

**Fig. 3:**
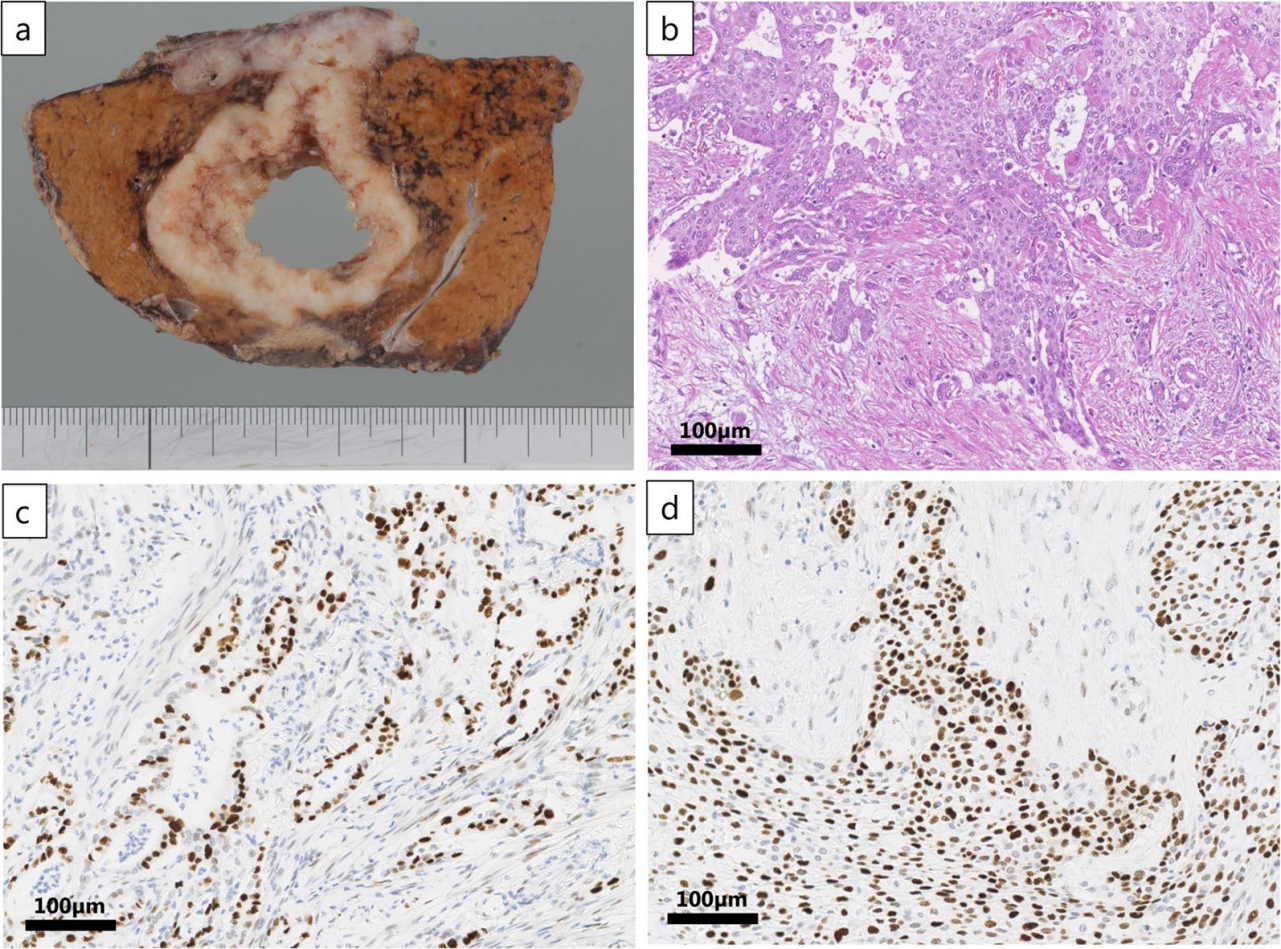
Findings of the excised liver specimen. **a** Gross examination of the excised specimen revealed a 6.0 × 5.5-cm white tumor with diaphragmatic invasion and a central cavity. **b** On pathological examination, the histology was consistent with squamous cell carcinoma with keratin-like material and necrosis. **c** Immunohistochemically, the nuclei of pancreatic adenocarcinoma were diffusely and strongly positive for p53. **d** Immunohistochemically, the nuclei of liver squamous cell carcinoma were diffusely and strongly positive for p53

A detailed review of all tissues from the previous surgery revealed no SCC-like tissue; similarly, no adenocarcinoma elements were identified in the liver biopsy specimen. Therefore, we decided to perform a partial hepatectomy with a presumed diagnosis of primary SCC that had developed after the surgery for the pancreatic cancer. At the time of surgery, the tumor had grown to 5.0 cm in size with some invasion into the diaphragm. Combined partial hepatic S5/6resection and diaphragmatic resection was performed by open laparotomy. The defect in the diaphragm was 5.0 × 4.0 cm and was closed with horizontal mattress sutures using monofilament sutures. Gross examination of the excised specimen revealed a 6.0- × 5.5-cm white tumor with diaphragmatic invasion (Fig. 3a). On microscopic examination, the tumor cells showed SCC histology with keratin-like material and necrosis (Fig. 3b). There was no obvious glandular duct formation. However, because primary hepatic SCC is extremely rare, we compared the pancreatic and hepatic lesions for *p53* expression and *KRAS* abnormalities. Immunohistochemically, *p53* was strongly nuclear stained in both lesions (Fig. 3c, d). *KRAS* genomic analysis of formalin-fixed, paraffin-embedded specimens was performed by polymerase chain reaction using reverse sequence-specific oligonucleotides (SRL, Inc., Tokyo, Japan). We found that the *Q61R* mutation was present in both lesions, and the diagnosis was therefore pure SCC metastasis from the pancreatic adenocarcinoma (Table 1).

**Table 1:** Comparison of *RAS* gene mutations in pancreatic and hepatic tumors

	Pancreatic AC	Hepatic SCC
KRAS codon 12	Negative	Negative
KRAS codon 13	Negative	Negative
KRAS codon 59	Negative	Negative
KRAS codon 61	Q61R	Q61R
KRAS codon 117	Negative	Negative
KRAS codon 146	Negative	Negative
NRAS codon 12	Negative	Negative
NRAS codon 13	Negative	Negative
NRAS codon 59	Negative	Negative
NRAS codon 61	Negative	Negative
NRAS codon 117	Negative	Negative
NRAS codon 146	Negative	Negative

*AC* adenocarcinoma, *SCC* squamous cell carcinoma

In a postoperative course, intraabdominal abscess at the resection site was observed, but it immediately resolved only with antibiotic therapy. The patient was discharged home on the 35th postoperative day and remained recurrence-free for 1 year. The benefit of postoperative adjuvant chemotherapy in these patients has not yet been proven, so it was not performed.

## Discussion

In this report, we experienced a case of pure SCC liver metastasis from pancreatic cancer without an SCC component in the primary tumor. Because we could not identify any histologic commonality between the liver tumor and the previous pancreatic cancer, we initially concluded that the patient had incidentally developed primary hepatic SCC after the pancreatic cancer surgery, although primary hepatic SCC is extremely rare [4]. Liver resection was performed, and examination of the excised specimen revealed pure SCC and no histological features in common with the previous pancreatic cancer (Fig. 3b). However, the *Q61R KRAS* mutation was identified in both the previous pancreatic cancer and the current liver tumor, leading us to conclude that the pancreatic cancer had mutated completely into SCC that formed a liver tumor (Table 1). Staining for p53 was also strongly positive for both pancreatic and hepatic lesions, supporting this conclusion.

In addition to adenocarcinoma and ASC, extremely rare cases of SCC in malignant pancreatic tumors have been reported [1]. Gruhl et al. [2] reported that ASC had a frequency of 0.70%, whereas SCC had a very low frequency of about 0.16%. The pathogenesis of primary pancreatic SCC is not clear, but several theories have been proposed, including the following: malignant transformation of squamous metaplasia secondary to chronic inflammation, an increase in only the SCC component without any adenocarcinoma component remaining from the ASC, and the presence of cancer stem cells with the ability to differentiate into both adenocarcinoma and SCC. In the present case, it is possible that the metastatic adenocarcinoma differentiated into adenosquamous carcinoma and only the SCC component remained in the liver, as in the second hypothesis, or that cancer stem cells were present in the metastatic lesion, as in the third hypothesis.

*KRAS* mutations are the most common genetic mutations in pancreatic cancer [5]. In pancreatic adenocarcinoma, *KRAS* mutations were found in 85.8% of cases, of which *Q61R* mutations were found in 1.2%, which was very rare with a frequency of about 1.0% of all PDAC cases. There are no reports about *KRAS* mutation in hepatic SCC [5]. On the other hand, in lung SCC, *KRAS* mutations were found in only 4.4% of cases, of which *Q61* mutations were found in only 3%, with a frequency of only 0.1% of the total cases [6]. Having the same gene mutation suggests that this liver tumor was highly likely a metastatic recurrent cancer rather than second primary cancers. It is reasonable to conclude that the pure SCC in the present case metastasized from the previous pancreatic cancer because of its common *KRAS* mutation. It was recently reported that *KRAS* gene abnormalities in ASC of the pancreas resemble those in adenocarcinoma [7]. It is possible that *KRAS* gene abnormalities in SCC of the pancreas are also similar to those in adenocarcinoma. In vitro experiments have shown that *Q61R* mutation in pancreatic cancer cells causes cancer cell proliferation but is sensitive to simultaneous inhibition of ERK-MAPK signaling and autophagy, and this is expected to have practical therapeutic applications [8, 9].

Surgical resection is the only curative option for primary pancreatic SCC, and no chemotherapy regimen has been established [3]. There have been reports of success using the combination of 5-fluorouracil and cisplatin, following the lead of reports describing the treatment of other SCCs [10]. One report described effective treatment with the combination of gemcitabine and radiotherapy [11]. Although treatments for SCC and pancreatic adenocarcinoma are possible options in cases such as ours, further accumulation of cases is needed to establish effective chemotherapy.

## Conclusion

This is the first reported case of pure SCC liver metastasis from pancreatic cancer without a squamous cell component in the primary tumor. Evaluation of *KRAS* mutations in both specimens was useful for diagnosis.

## Data Availability

Not applicable.

## Abbreviations

SCC: Squamous cell carcinoma
CT: Computed tomography
MRI: Magnetic resonance imaging
ASC: Adenosquamous carcinoma
